# RETRACTED: Dewi et al. Efficacy of Intermittent and Continuous Subglottic Secretion Drainage in Preventing the Risk of Ventilator-Associated Pneumonia: A Meta-Analysis of Randomized Control Trials. *Medicina* 2023, *59*, 283

**DOI:** 10.3390/medicina59111900

**Published:** 2023-10-26

**Authors:** Yulis Setiya Dewi, Hidayat Arifin, Rifky Octavia Pradipta, Arina Qona’ah, Rosita Rosita, Cindy Nanda Giatin, Amel Dawod Kamel Gauda

**Affiliations:** 1 Faculty of Nursing, Universitas Airlangga, Surabaya 60115, Indonesia; 2 School of Nursing, College of Nursing, Taipei Medical University, Taipei 110, Taiwan; 3 Palembang MediRose Publisher, Palembang 30154, Indonesia; 4 Maternal and Newborn Health Nursing, College of Nursing, King Saud bin Abdulaziz University for Health Sciences (KSAU-HS), Ministry of the National Guard, Riyadh 11173, Saudi Arabia; goudaa@ksau-hs.edu.sa; 5 Department of Maternal and Newborn Health Nursing, Faculty of Nursing, Cairo University, Cairo 11562, Egypt

The journal retracts the article entitled “Efficacy of Intermittent and Continuous Subglottic Secretion Drainage in Preventing the Risk of Ventilator-Associated Pneumonia: A Meta-Analysis of Randomized Control Trials” [1].

Following the publication, concerns were brought to the attention of the Editorial Office regarding a number of irrelevant studies that were included in the analysis of this systematic review and meta-analysis [1].

Adhering to our complaint procedure, an investigation was conducted by the Editorial Office and Editorial Board, which confirmed that a significant number of randomized control trials reviewed were not eligible to be included in the analysis of this systematic review and meta-analysis. Specifically, the inclusion of Chai et al. [2], Chow et al. [3], Philippart et al. [4] and Qiao et al. [5] was considered to have limited relevance to the main topic of this systematic review and meta-analysis, and consequently, was judged to sufficiently compromise the overall findings to a degree that the findings could not be relied upon. Moreover, as a result of this methodological flaw, erroneous analysis and interpretation of the findings was performed. The article is therefore retracted.

This retraction was approved by the Editor-in-Chief of the journal Medicina.

The authors did not agree to this retraction.
